# THE PAINS OF PARAMILITARISM: The Latent Criminogenic Effects of Exposure to Paramilitary Violence Among Young Men in a Post-Conflict Society

**DOI:** 10.1007/s40653-023-00516-2

**Published:** 2023-01-13

**Authors:** Colm Walsh, Twylla Cunningham

**Affiliations:** 1 Queen’s University Belfast, Belfast, United Kingdom; 2 Probation Board for Northern Ireland, Belfast, United Kingdom

**Keywords:** Conflict, Crime, Northern Ireland, Offending, Paramilitarism, Prevention, Violence, Young Men

## Abstract

**Purpose:** Whilst most people who experience adversity recover, there is a cumulative body of evidence that illustrates that the effects can be long lasting, and can even become debilitating over time. Links have been made between traumatic distress, mental health disorders and disturbances in behavioural and emotional regulatory systems that may in context elevate the risk of offending. Despite the burgeoning evidence around the criminogenic effects of adversity, few studies have examined the traumatic effects of paramilitary related adversity in the context of post-conflict Northern Ireland. **Methods:** With reference to DSM-V PTSD diagnostic clusters, the aim of this study was to explore the latent impact of adversity and latent trauma among justice involved young men and identify potential criminogenic effects of exposure to paramilitary related adversity. **Results and conclusions:** This study found that across the sample, young men had self-reported to have experienced significant adversity, including violent victimisation. Exposure to paramilitary adversity often began during early adolescence. The participants described symptoms that were consistent with clinically diagnosable disorders such as Post-Traumatic Stress Disorder. Despite this, there appears to be a paucity of trauma screening and assessment, and few supports that victim could benefit from. In the absence of appropriate and evidence-based supports, many young men appear to find other (and more maladaptive) ways to cope. This exacerbates the risk of interfacing with the justice system and may even contribute towards a deterioration in wider psycho-social outcomes. Implications for practice are discussed.

## Introduction

### Adversity and Trauma and Cumulative Effects

First articulated by Feletti and colleagues in 1998, the concept of Adverse Childhood Experiences or *‘ACEs’* provided novel language that has contributed towards a greater appreciation of the impact of early adversity and psychological trauma on future outcomes (Devaney et al., 2020). In the multi-disciplinary study of youth violence prevention, the concepts of adversity and trauma have become almost ubiquitous *(*Malvaso et al., 2021). Adversity is something that most of us experience (Carter et al., 2021), with estimates that one-in-two people in the United Kingdom experience it during childhood (Bellis et al., 2014). Whilst the experiences may be difficult, the effects are often short-lived. For others however, the impact can be more enduring (Davidson et al., 2010; Cecila et al., 2017), and can result in the onset and persistence of psycho-social complexities (Cloitre et al., 2009; Lawson and High, 2015). This is particularly the case when multiple adverse life events are experienced (Farrell & Zimmerman, 2017). Trauma can therefore be thought of as the psychological responses to these adverse experiences which commonly include exposure to (or the threat of) interpersonal adversities such as physical and sexual abuse, maltreatment and community violence (Finkelor, 2021; Malvaso et al., 2021).

Studies have identified that a link exists between traumatic distress, mental health disorders and disturbances in behavioural and emotional regulatory systems (see for example, Bremer and Vermette, 2001) that may in context, elevate the risk of offending (Widom, 1989; Ardino, 2012; Malvaso et al., 2021). Whilst it is true that not all of those who are traumatised go on to offend, it is also true that there are concentrated levels of trauma within justice involved populations (Maschi & Bradley, 2008). Interpersonal adversities appear to be particularly salient (Finkelor et al., 2021; Nöthling et al., 2016; Hamby et al., 2021), with exposure to community violence associated with elevated stress responses (Buka et al., 2001; Gaylord-Harden, So and Bai, 2017). Greater proximity is associated with greater levels of stress (Fowler et al., 2009), as well as with elevated risk of violent offending (Walsh et al., 2021). There is also increasing evidence that poly-victimisation (exposure to different types of violence across systems) exacerbates risk of psycho-social harm even further (Turner et al., 2010; Farrell & Zimmerman, 2017; Ahlin & Antunes, 2021), contributing to trauma related disorders.

One of the most widely studied trauma related disorders is Post Traumatic Stress Disorder (PTSD) (DSM-V, 2013; Ateka et al., 2018). PTSD is a complex, and sometimes chronic mental health disorder that causes substantial distress, that without appropriate supports can interfere with social, emotional, behavioural and educational functioning (Braga et al., 2017; Trickey et al., 2010; Malvasso et al., 2021). Whilst the aetiology of psychological distress following trauma is not well established (Maschi & Bradley, 2008), peri-trauma factors such as the presence or absence of social supports may remediate or elevate distress. Without social supports, the onset and maintenance of disturbances such as PSTD is often increased (Guay et al., 2006; Maschi & Bradley, 2008; Trickey et al., 2010). This may be even more elevated when those supports, who are expected to prevent harm, cause or facilitate the harm (Tirone et al., 2021). Defined as betrayal trauma (Freyd, 1994), the relational proximity of perpetrators to the victim is uniquely predictive of PTS severity (Kelley et al., 2012).

Whilst only around 10% of those exposed to adversity will develop clinically diagnosable symptoms, for those whose symptoms are more persistent, the effects can be profound (Maschi and Bradely, 2008; Horn et al., 2016; Aebi et al., 2017). *DSM*-V outlines the key criteria for a PTSD diagnosis. Four symptoms clusters form the basis for making clinical decisions (Weathers et al., 2013). Despite the prevalence of exposure to violence among young people in Northern Ireland (Bunting et al., 2020), many youth with clinically diagnoseable symptoms are not routinely screened or assessed for PTSD (Duffy et al., 2021). Despite the paucity of clinical assessments, trauma affected individuals may still present with complex symptoms such as re-experiencing, negative emotions and mood, hyperarousal and avoidant symptoms, all of which are consistent with Post Traumatic Stress.

#### Re-Experiencing

Intrusive re-experiencing is a core symptom of PTSD, often involving intrusive images, flash-backs and nightmares (American Psychiatric Association, 1994; Hellawell & Brewin, 2002). Individuals with PTSD sometimes re-experience physiological sensations or emotions that were associated with the traumatic event without a recollection of the event itself without being able to put it into context. In this sense, the cues may only be indirectly related to the traumatic event (Charney et al., 1993; Ehlers and Clark, 2002) People with PTSD are usually unaware of these triggers, so intrusions appear to come out of the blue and increase levels of distress (Ehlers et al., 2002, 2004).

#### Negative Emotions and Mood

This can include a range of issues such as a sense of blame, to diminished interest in activities. It can also affect episodic memory around the experience/s (Tirone et al., 2021). PTSD can also co-occur with other mental health issues (Cohen et al., 2010) such as anxiety and depression (Foa & Rothbaum, 1989; MacDonald et al., 2010), as well as substance use disorder (Brewin et al., 1996). Indeed around half of those diagnosed with PTSD have comorbid issues such as major depressive disorder (Contractor et al., 2014), adding to the clinical and diagnostic challenges (Nöthling et al., 2016).

#### Hyperarousal

Hyperarousal could be particularly salient in the field of violence prevention (Fowler et al., 2009). Some individuals may lose the capacity to differentiate between safe and threatening environments, presenting with externalising difficulties as they respond threats when in fact, no such threat exists (Greenwald, 2002; Silvern & Griese, 2012; Malvaso et al., 2021).

#### Avoidance

Another common symptom cluster of PTSD (indeed an early predictor of the onset of PTSD (APA, 2013) is the psychological and physical avoidance of people/places/things that serve as reminders of the traumatic event (Morris, Salvovskis, Adams, Lister, & Meiser-Stedman 2015). From a criminological perspective, this avoidance in response to psychological strain (Agnew, 2001), is considered to be a form of corrective action. This *‘escape-avoidance’* (Farrell & Zimmerman, 2017), whilst likely to be adaptative in the immediacy of exposure, in the long term, can become maladaptive (Gaylord-Harden, So and Bai, 2017), and the emotional numbing that often accompanies such strategies may partially explain why some of those who have been violently traumatised go on to engage in violence themselves (Tolan & Gorman-Smith, 1998; Fowler et al., 2009). Indeed research has found that poly-victimisation predicts both increased emotional numbing as well as increased aggression (Turner et al., 2010; Allwood et al., 2011; Farrell & Zimmerman, 2017).

Despite the paucity of clinical screening for PTSD among trauma affected children and young people in Northern Ireland, the identification of individuals presenting with difficulties consistent with symptom clusters could enable services to connect those in need of therapeutic support to those supports (Duffy et al., 2021).

The Probation Board for Northern Ireland (PBNI) is a non-departmental public body that has the aim of enabling safer communities and reducing re-offending. The *‘Aspire’* project was developed by the PBNI to supervise and support justice involved young men contemporarily at risk of paramilitary related harms (NISRA, 2019). Since its inception in 2017, the project has provided mentoring and employment support, housing advice and mediation services to young men aged 16–25. Whilst the criminogenic effects of adversity within the justice involved population are now well established (Ardino, 2012; Gaylord-Haden, So and Bai, 2017; Walsh, 2019), few studies have examined the traumatic effects of paramilitarism within the justice involved population in the context of post-conflict Northern Ireland.

With reference to DSM-V PTSD diagnostic clusters, the aim of this study was to explore the latent impact of adversity and latent among justice involved young men and identify potential criminogenic effects of exposure to paramilitary related adversity.

## Method

The team were particularly concerned with the ideas that can embody exposure to and recovery from violent trauma (Metzler et al., 2021). A purposive sample (Patton, 2015) of young men aged between 18 and 30 were recruited. The only inclusion criteria were that participants were involved with the *‘Aspire’* project which was designed by PBNI to engage young men at risk of paramilitary related harm. Potential candidates were first identified through their routine meetings with their probation officers. Aspire probation officers (PO’s) were provided with a research information sheet and consent form. Both the information sheet and consent form made it clear that participation in the study was voluntary. POs were asked to read the details to the young men. Once advised of the study, the young men were given a one-week *‘cooling off’* period. At the following meeting with their PO, the young men were asked whether they would like to opt in to the study or not. The consent forms for those who consented to engage were forwarded to the research team. They were contacted by the researcher to arrange the interview. Those who were considered by the PO and/or the researcher not to have the capacity to consent were excluded. This did not apply to the current study. Those who were unable to converse in English were also excluded. This did not apply to the current study. A semi-structured interview schedule was developed and an interview protocol was implemented. At the outset of each interview, the interviewer confirmed that each participant understood the nature of the study and asked the participant to confirm that they consented to particulate. The interviewer also reminded the participants of their right to withdraw. The interview schedule consisted of three broad themes within which there were a range of illustrative questions and prompts: ‘about you’ (e.g., tell me a bit about yourself-what do you like to do?); ‘about living in your community’ (e.g., how active are paramilitaries in your community?) and; ‘about your supports’ (e.g., have you ever had a mental health diagnosis?) Audio-recorded interviews were undertaken by the first and second author, each lasting between thirty and sixty minutes; with the average being approximately forty minutes.

### Ethics

Ethical approval for the study was granted by both the PBNI research ethics panel and from Queen’s University Belfast School Research Ethics Committee (SREC).

### Data Analysis

All interviews were transcribed verbatim, and analysed inductively using thematic analysis (Braun & Clarke, 2019). To improve validity, both researchers worked independently, choosing three randomly selected transcripts, and agreeing on codes (Guest et al., 2012). Three meta-themes and forty-one operational themes were identified as part of the wider study. The meta-themes included: latent paramilitary related psychological trauma; trust in services and; male norms. The meta-theme that is discussed in the current paper is latent paramilitary related psychological trauma in post-conflict NI.

## Results

### Descriptive Data

Twenty-eight young men involved with PBNI Aspire project consented to engage in interview and 66.6% (n = 18) of those who consented to participate were ultimately interviewed. Reasons that others did not engage in the interview included: sickness (7.1%, n = 2); return to prison (3.6%, n = 1); crisis situation (3.6%, n = 1); employment (3.6%, n = 1) and; loss of contact (17.9%, n = 5). Table 1 provides an overview of the sample characteristics. Just over one-third (33.3%; n = 6) of the sample were from a Catholic/Nationalist/Republican (CNR) area, whilst 66.7% of the sample identified as being from an area characterised as being Protestant/Unionist/Loyalist (PUL). The sample was drawn from communities across Northern Ireland, however, the data is not regionally disaggregated.

**Table 1:** Sample characteristics

	n/%	
N (sample)	18	
Community Background	PUL = 66.6	CNR = 33.3
**Evidence of at least one adverse experience**	**88.9**	
**Mental health issues**	**94.4**	
**Mental health Diagnosis**	**50**	
**Substance related issues**	**100**	
**Paramilitary threat/intimidation**	**94.4**	
**Age at first paramilitary exposure**	**13**	
**Victim of paramilitary violence**	**100**	
**Trauma screen/support undertaken**	**11**	
**Informal support network available**	**27.8**	

## Narrative data

### Social Ecological and Systemic Adversity

Across this sample, it was evident that adversity was endemic and chronicity played an important role in the onset of difficulties. An overwhelming number of participants described difficult home lives during childhood, with caregivers often having their own complex psycho-social issues themselves. Several described experiences of maltreatment illustrated by time spent in state care, and characterised as transitionary and unstable.

Well I’ve been in and out of prison since I was thirteen, because I’ve been in and out of hostels… I just kept getting put into foster care, and because I kept running away from the house and all the police were out looking me and all and they were asking me what was going on, and I ended up telling them (5032)

These transitions were illustrative of instability within young men’s family lives, a factor that appeared to contribute towards greater time spent in the community. A range of specific family contexts characterised the more general pattern of a push out of the home and a pull towards wider contextual harms.

Aye. I used to flipping go out and all, stay with all my mates and that there and not come home at all. (2033)

…my mum was an alcoholic and stuff so I wasn’t always in the house, do you know what I mean? I was always out. Then obviously I became aware of people and I started to get to know the wrong people and just fell into the wrong crowd (6027)

For several participants, home was not only stressful, but dangerous. Young men described living in situations where they were at risk, as were those around them.

I was in, my mum was round with like violent boyfriends and, they were doing stuff and then social services took me off my mum…(5032)

Loss appeared almost endemic-from the loss of loved ones following relationship breakdown, to the death of parents and siblings-often in very difficult ways. From the perspective of these young men, this loss preceded years-even decades of socio-emotional issues.

Aye, I had a lot of problems and everything over the years like. But I lost my ma too whenever I was only three months old, and my granny took me. Two of my granny’s kids committed suicide. My mum and her wee brother… She was only nineteen and her brother was only fifteen. (6023)

These difficulties were not limited to the family environment-nor temporally constrained to childhood. Young men described difficulties as being nested in their social ecology. School, a place where orthodoxy and established norms dictate that they would spend a significant part of their time-was a place of tension and trouble. Young men consistently referred to absence reduced attainment.

Obviously I didn’t really get much education when I was younger. I was just always in trouble all the time and sent home (2033)

Aye. I don’t have no GCSEs like, I have like Level Twos…I didn’t do them, no. In fifth year, I got to fourth year but then, I was always messing about in school so they took me out of the normal class in fourth year, and in fifth year I had to go into like, there was a group of seven and we just done Level Twos and like a Duke of Edinburgh award and stuff like that. (2037)

For some, the lack of engagement in education was related less to disciplinary practices than a function of their home life. Within the context of transient and unstable home-lives, education was rarely the priority.

I came back when I was eleven, and then went to primary school for a wee while, then moved into foster care then, and then after foster care then when I got out in the second year I moved back in with them again, with my ma so I did. So I didn’t do any education or nothing like that like. (2025)

A natural consequence of school absences was increased time spent un-supervised in the community, and with others who were equally unsupervised – a consistent predictor of increased risk of violent crime.

Na mate-we kinda done our own thing. You just would have went out when you got up-met up somewhere and walked about the estate until you went home. Sometimes it was Baltic like-but sure what else would you be doing? (5032)

This combination of reduced school engagement combined with reduced ‘guardianship’ or informal social control within the context of communities that were characterised as being less safe, exponentially increased the risk of the young men being exposed to potentially traumatic events.

[my friend] rung me one day and says *‘come up to my house’*. I went up to his house and found him hanging, and from that there my head started going… I started taking more coke, taking more steroids. (3512)

A minority of young men recalled navigating new social spaces that were neither well understood nor safe. Living in unfamiliar areas as their family situation changed periodically, created new challenges.

No. No, I’m not from here. Well you could say I’m not really from anywhere. I was born somewhere obviously, but we moved that much to be honest and I lived with that many different people, I never really felt like I lived anywhere-you know? I remember walking about at the weekend and you weren’t really sure where to go or where not to go (2026)

### Violence and Paramilitarism

One of the most consistent sub-themes within these narratives was the high levels of violent trauma and the enduring presence of paramilitary groups.

Yeah, yeah they’re very active, very, very active…And if you just read the papers like, the papers tell a lot of lies, but it more or less gives you a hint, do you know what I mean? (3512)

In fact, 94.4% of the sample demonstrated some evidence of both paramilitary threat/intimidation as well as paramilitary related violence. At the same time, there was a level of tolerance, an acceptance of the status quo, at least in part because few could see feasible alternatives. Despite what most young men witnessed, it was caveated with sentiments akin to *‘what can you do?*’

Aye, because I’ve had all sorts of death threats, do you know what I mean? But there’s nothing you can do. (4034)

On average, participants were 13 years old when they reported being first exposed to paramilitaries. However, the ages of first exposure ranged being between 11 and 17 years old.

I seen a lot of fighting and stuff like that there when I was younger. It wasn’t very nice like - it wasn’t very nice seeing that sort of stuff. I’ve been under threat since I was like thirteen or fourteen. (3512)

The most common type of exposure was physical violence. Most described- often in graphic detail - the level of violence inflicted on them and others around them.

I got bottled over the head and all twice as well, and hit over the legs with hammers and then jumped on (3028)

Every one of my mates have all been shot, every one of them. (6023)

The first night the gun jammed in the back of my head. Then the second night they came back and tried to shoot me (4024)

Perpetrators were often known to the victims. They may have even been family. Indeed there were examples where family members (for reasons unknown) facilitated some of the violence.

I think the first time there was three and then after that there was always five of them…one of the people that organised and done the whole thing was their uncle…My da would’ve drank with them as well all the time. (3028)

Compliance, tacit approval and resignation to the dark realities of community life all featured in these narratives as they described the harms and justifications in equal measure. As one young man commented, *“There was nothing he could do. Just pretty much said ‘it’s your own fault’” (3028).* For several, there was a fatalism that permeated their outlook. If fear could be tempered, then the most traumatic of possibilities could be embraced. As another participant suggested, tempering fear meant accepting the inevitable: *“…I wasn’t afraid like. I knew it was coming. It was just a matter of time (2025)”.* This also reflected a general sense of level of deservedness or self-blame. Many young men believed that their victimisation was justified using the following logic: *I live in an area where those engaged in [some] crime are punished violently; I engaged in [this form of] crime on the understanding that the potential for violence was likely; I therefore deserve the punishment.* Exposure to paramilitary harm also included property damage, extortion, theft and the intimidation of family members.

They demanded money and I walked away from it all, and then they tried to break into my flat and give me a beating. They spray painted my name on the walls; they wrecked my car; they intimidated my family. (6027)

A couple came in covered up and took my car and a couple of pound…I won’t give them anything. I have nothing to give them no more… (4022)

Such examples reflected a wider pattern of exploitation. Whilst the immediate experiences were often short-lived, such exposure had a lasting effect. Those in financial hardship, as well as those with substance abuse issues found themselves to be particularly vulnerable to a range of harms.

I needed money. When I was about nineteen or something [the paramilitaries] were giving me about a grand or something, two grands’ worth of cannabis…And it got too much for me and I ended up in hospital. (2033)

### Traumatic Effects

Many described post-trauma symptoms consistent with the re-experiencing cluster. Young men described how traumatic events continued to be experienced in various ways in the weeks, months, even years that followed. Symptoms were by no means limited to the re-experiencing symptom cluster. Most of the young men described mood and anxiety related issues consistent with exposure to violent trauma, with many young men describing how they had tried to find ways of coping with the psychological strains.

Anxiety and stuff like that there, because that there’s why I started taking drugs, because I always had anxiety. Like even when I was in school I took a panic attack (4018)

In line with DSM-V criteria, self-blame was particularly strong across the sample. As one participant noted with some logic, *“it was nobody else’s like…it’s not right. But at the same time my behaviour wasn’t right either (*2026)”. But this also impacted on young men’s sense of safety, with many being hyper-aroused to social stimuli.

You just don’t feel safe. How can you. You hear things and you turn round expecting it to be someone out for you. Even in the house-you hear a bang and you jump. You think *‘they’re coming’*. (4022)

Whilst some young men were appreciably frightened of the prospect of harm, even historical threats contributed towards psycho-somatic responses consistent with clinical disorders such as PTSD.

You’re always watching over your shoulder…when I was like sixteen, they gave me that death threat…they seem to be leaving me alone, but you’re still waiting…(13020)

What was consistent were the reports of involuntary emotional and behavioural responses to social stimuli such as noises across a variety of environments. One young man noted that even with *“the slightest noises I was hiding under the bed…I feared them like, aye…plenty of times I’ve heard bangs and thought I was getting killed”* (4022).

Another alluded to the defensive, but functional behaviours that were as much about reducing heightened emotional states as they were an attempt to pre-empt threats against them.

I was so stressed. I was convinced that the [paramilitaries] were going to come through my ma’s front door, you know, to get to me. It obviously never happened, but like I was convinced in my head at some stage it would happened and I was running about paranoid, and obviously because of the drugs I was taking I was paranoid too, and then I started like carrying knives and stuff like that there. (4018)

Consistent with literature, there were numerous accounts of young men misinterpreting social cues-perceiving there to be threats when in fact no such threats were present. The effects of such misinterpretations appeared to extend beyond the young men themselves to directly affect others around them. Indeed, there were examples of bystanders unwittingly triggering these states and finding themselves the victim of verbal and/or physical violence.

Like you know the way somebody just looks at you strange like?...I go to start up the big row with them like…Just because I think they’re trying to make a c**t out of me inside my house and in front of my family like…just by looking at me. (6030)

### Avoidance

Avoidant behaviours were described in several ways. For example, some young men described a reluctance to engage with, or access services. This included medical services.

I refused it. I have scars and all that I could’ve got stitched and now they’re just, because I didn’t get medical attention…I didn’t want the talk and investigations. I just didn’t want nothing to do with it. I just wanted to get home and away into bed.

The short term solution was to find alternative ways of avoiding talking about the incidents-blocking the thoughts from themselves.

I ended up getting attacked and I ended up losing the sight in my left eye. So after that there I ended up going back to my ma’s and like just didn’t really leave the house, you know? Just stayed in every day just taking prescription drugs and cannabis and alcohol and stuff like that there. (4018)

For many others, this avoidant behaviour was not limited to the hours, or even days following the event, but extended into months and years Indeed, there were examples of such behaviour affecting many areas of the young men’s social and work lives as time progressed .

I didn’t like leaving my comfort zone [my area] if you know what I mean. I sort of stayed in the area, like I wouldn’t like travel anywhere else. (6027)

Several of the young men also described making material changes to patterns of behaviour, with some consciously avoiding routine activities and actively avoiding certain areas.

I don’t really go anywhere at the minute. I only leave the house whenever I have to. If we’re going to the shop or I’m going to get my hair cut or stuff like that. (4018)

### Lack of Assessment and Supports

Despite the ubiquitous exposure to a range of harms and evidence of mental health issues, the majority of individuals (94.1%, n = 16) reported no mental health assessment, and 89% (n = 16) reported no trauma screening or assessment process. There was evidence that only in a minority of cases were post-traumatic stress symptoms appropriately assessed by clinicians.

I’ve always had like mental health problems but I’d never really had them addressed. (4018)

There was no evidence of appropriate trauma specific treatment being facilitated to ameliorate the presenting symptoms. At best, young men were referred for generic counselling.

I’ve never been like properly diagnosed…I just need to know if I have, or if I’ve got PTSD…Obviously from losing the sight in my left eye…and then like a few weeks before that there I got attacked in a house… and when I got attacked I got a knife put to my throat and stuff like that there, do you know what I mean?...there’s a whole lot of things that’s happened to me…(4018)

There were some examples of the young men seeking more adaptative ways of coping, but in these cases their attempts were counterbalanced by the systemic challenges they faced. One young man had contacted a specialist mental health NGO. After gathering the motivation, navigating public transport, and asking to speak with someone at the NGO directly, he was told that he could not benefit from their supports. Without appropriate signposting, he returned home.

So, I tried to go to them by myself, but I’ve been told that they’re not allowed to take me…I don’t know what way to explain it, but yeah I went to numerous places and they all turned me down. (2026)

Given the lack of trauma specific assessments and supports, young men found other ways of coping with their distress. Most described an intimate, but difficult and prolonged relationship with substances.

I went to drugs and that there to blank things out…I was really bad on them there. I just blanked things out, and then ended up taking seizures on the buds (2033)

Drug use was a form of self-medication that appeared to mask memories of adversity and a range of trauma related symptoms.

As I got older like it was harder to deal with it, so then I started smoking weed. One of my mates handed me a joint because I was crying at my mum’s grave one day and he handed me a joint and I started smoking it, and then- just boom- I switched off and started laughing. And then maybe a year or a couple of months, a couple of months to a year, maybe a couple of years after it I started sniffing coke like. (6030)

As well as helping young men avoid emotional distress, it also dampened the effects of hyper-aroused states. Given that many of this sample believed that they could be still under threat (and several relived their previous experiences), drugs enabled some level of functioning in the community.

So like I’ve always had like problems with people in that area, even when I was living down there, so I think that there’s the reason why I was taking so much drugs as well, do you know, because obviously I got like when I was, I couldn’t walk about unless I was off my head. (4018)

In retrospect, several participants reflected on the damage that this caused and the difficulties that were exacerbated by their chronic self-medicating. Issues ranged from relationship difficulties, unemployment, entry into care to violent offending.

Yeah. Yeah, yeah – with the drugs there was a lot of fighting. That was definitely the way I coped. Like my childhood, I grew up in care and stuff man, do you know what I mean? Yeah, so that’s how I coped, I just took loads of drugs and blanked out. Like at age eleven and twelve I was taking ecstasy man, you know, it’s documented. Social services have a document of three-to-four-day benders at twelve years of age on E’s [ecstasy]. (3020)

A minority found other, but no less problematic ways of coping. A number of young men alluded to a history of paramilitary involvement in their own families. In these cases, young men in crisis saw opportunity. For some, familial attachments could increase the chances of avoiding the trauma of violence in the first place.

We were all threatened and [inaudible], but I just never went and met them. Most of my mates all went and met them, and just my family background they weren’t really able to shoot me, do you know what I mean? (6023)

For others, joining the ranks of those groups was believed to be preferable to the alternative of further violence. Membership offered the promise of protection and at the same time-equated to control. The nature of living in difficult circumstances was that some of these young men believed their choices to be so limited that they found some solace in *‘signing up’.*

[going ‘under the belt’] means you’re under their control now like, and then you’re under the belt, it’s like you’re under their wing, so if like another organisation comes near you and asks you for anything you just say [the name of the group] (4022)

## Discussion

Understanding the context of violence and its harms is important for prevention (Ahlin & Antunes, 2021). Decades of research have illustrated that exposure among the most vulnerable groups begins early, and often in the home (Widom, 1989; Bellis et al., 2014), later extending into school (Ozer, Lavi, Douglas & Wolf, 2017), and into the community (Fowler et al., 2009). Indeed, early adversity in the home appeared to be related to greater time spent in communities among this sample, which elevated the risk of other harms (Sampson & Groves, 1989; Akers & Sellers, 2004; Blum & Naranjo-Rivera, 2019).

This exploratory study uncovered a rich narrative that illuminates the range of nested ecological stressors placed on this sample (Davidson et al., 2010; Milaniak & Widom, 2015; Ahlin & Antunes, 2021), as well as the complexity of paramilitary harms that continues in post-conflict society NI. Consistent with previous research, it was not only evident that paramilitaries have an enduring presence in some communities (Walsh et al., 2021), but that the traumatic effects of their presence on communities has been under-evaluated. Consistent with previous studies in the Northern Ireland context, (see for example Duffy et al., 2021), many of these young men, despite experiencing multiple and often violent adversities during childhood had not been screened, assessed or treated for trauma related conditions.

Whilst adversity was common, violent adversity was almost endemic across the sample (Hamby 2021), with exposure beginning during childhood. Early and persistent exposure has been connected with the onset of range of difficulties, including criminal behaviour (Milaniak & Widom, 2015; Sharkey, 2018). What was interesting in these narratives was that perpetrators were often known to the victims (Elsaesser, Kennedy & Tredinnick, 2019). They may have even been family. Indeed there were examples where family members (for reasons unknown) facilitated some of the violence on these young men, reflecting what Tirone et al., (2021) refer to as *‘betrayal trauma’.* The impact of such adversity was described in detail by the participants, much of which was consistent with diagnostic criteria for stress related disorders such as PTSD. Although clinical tools were not used during the interviews, the interviews elucidated underlying difficulties that support the need for clinical follow-up.

The findings confirm previous research on the enduring and long arm of violent adversity (Bellis et al., 2014; Sharkey 2018). In particular, the impact of poly-victimisation within the justice population (Fowler et al.,. 2009; Ardino 2012; Farrell & Zimmerman, 2017; Blum-Naranjo-Rivera, 2019; Malvaso et al., 2021). In contrast to previous studies however, this research explored the context in which violence was experienced (Ahlin & Antunes, 2021). Whilst the aim of the study was not to assess or to screen for elevated stress symptoms, there appeared to be high rates of exposure to a range of early adversities across this sample along with latent trauma which could contribute towards offending behaviours. This exploratory qualitative investigation suggests that self-reported exposure to higher harm violence such as paramilitarism may contribute towards elevated post-traumatic stress symptoms, maladaptive coping strategies such as substance use, and increased risk of offending among a sample of young men living in post-conflict Northern Ireland.

Consistent with diagnostic criteria, self-blame and deservedness emerged as a key theme across the sample (Cohen, 2010). Many of these young men perceived that the violence that was inflicted upon them was proportionate and just. Whilst seen as a normative response to their own behaviour, few recognised that such behaviours could have been reflective of trauma responses. Psycho-education at an individual and community level could increase understanding around the ways that trauma may present .

Other stress related symptoms were also evident, including the reliving of traumatic experiences (Ehlers, Hackman and Michael, 2004) mood and anxiety-related difficulties and hyperarousal. Hyperarousal is particularly salient in study of violence (Fowler et al., 2009) and could be a target area in the prevention of violence related injuries (Allwood et al., 2011). Without appropriate supports, young men found succour in particular, coping cognitively and emotionally through the use of substances (Morris et al., 2015). It could be reasonably assumed that with appropriate supports, many victims of violence may avoid the criminogenic effects of chronic drug use.

There are a number of other practical implications. Whilst these data reinforce the need for early screening (Morris et al., 2015; Finkelhor, 2018; Duffy et al., 2021), there is a pressing need to establish how best to screen for potentially traumatic stress (Silva, 2004). What we do know is that left undetected (and therefore left untreated), PTS symptoms are likely to persist (Kerig et al., 2012; Guay, Beaulieu-Prévost, Sader and Marchland, 2019) with estimates that up to 30% of cases symptoms can last for five years or more (Smith et al., 2007)- frustrating given that we know that there are highly efficacious models of treatment/intervention? (Cohen et al., 2010; Duffy et al., 2021). However, if services are not better equipped to identify those who are at potential risk then services will not be in a position to connect service to need (Zammit et al.,. 2018). There is a need to understand in order to respond (Platt and Turney, 2014; van Vugt et al., 2014). This is particularly the case for understanding and responding to the trauma of young men (Jaycox, Marshall and Schell, 2004), and those living in societies defined as having elevated rates of violent conflict (Walsh and Gray, 2019). This exploratory study elucidated the range of latent adversities and trauma symptoms experienced by justice involved young men in Northern Ireland. Consistent with previous studies, the findings point to the need for earlier and more routine mental health screening (Duffy et al., 2021) and suggests that in addition to identifying those with elevated stress symptoms, the provision of evidence-based trauma treatments could reduce the criminogenic effects of violent and paramilitary related adversity.

## Limitations

There were several limitations to this study. Firstly, as a qualitative investigation, the sample may not be representative of all justice involved young men in Northern Ireland and therefore caution should be applied with generalisation some of the findings. However, one of the key findings was that justice involved young men in this study had experienced a range of adversities including violent and paramilitarily related adversity. The cumulative body of international evidence suggests that this is predictive of the onset of a range of difficulties including criminal behaviour-something which this data supports. Secondly, the participants were recruited via the probation officers. Despite the efforts made to reduce perceived pressure, some young men may have felt compelled to engage whilst others may have opted out of the study given the association with their probation officers. Thirdly, there was some potential that participants would not actively engage in the interview or fear that their responses would be reported back to their probation officers. However, the process for dealing with disclosures was transparently explained in the information sheet and the limitations of trust were verbally outlined at the outset of each interview. Given the findings, there is little concern that relevant details were withheld.
